# Effectiveness of pulmonary rehabilitation for patients with asthma: study protocol of a randomized controlled trial (EPRA)

**DOI:** 10.1186/s12890-017-0389-3

**Published:** 2017-03-09

**Authors:** Konrad Schultz, Hildegard Seidl, Danijel Jelusic, Rupert Wagner, Michael Wittmann, Hermann Faller, Dennis Nowak, Michael Schuler

**Affiliations:** 1 Klinik Bad Reichenhall, Center for Rehabilitation, Pulmonology and Orthopedics, Bad Reichenhall, Germany; 2 Institute of Health Economics and Health Care Management, Helmholtz Zentrum München (GmbH) - German Research Center for Environmental Health, Comprehensive Pneumology Center Munich (CPC-M), Member of the German Center for Lung Research (DZL), Munich, Germany; 3 Department of Medical Psychology and Psychotherapy, Medical Sociology and Rehabilitation Sciences Section, University of Würzburg, Würzburg, Germany; 4 Institute and Outpatient Clinic for Occupational, Social and Environmental Medicine, LMU University of München, member DZL, German Centre for lung Research, Munich, Germany

**Keywords:** Pulmonary rehabilitation, Asthma, RCT, Longitudinal study, Asthma control, ACT, Inpatient rehabilitation, Randomized controlled trial, Longitudinal, Economic evaluation

## Abstract

**Background:**

Asthma patients are enrolled in multimodal pulmonary rehabilitation (PR) programs. However, available data for the effectiveness of PR in asthma are sparse. Therefore, the primary aim of this randomized control trial (RCT) is to evaluate short-term (end of rehabilitation) and intermediate-term effectiveness (3 months after rehabilitation) of PR for patients with asthma regarding asthma control (primary outcome) and other outcomes. Secondly, moderator effects of gender, age, baseline asthma control, quality of life, and anxiety will be examined. Thirdly, a longitudinal follow-up study will explore the course of the outcomes over one year and the annual costs.

**Methods:**

The EPRA study is a single-center randomized controlled waiting-list trial in the Bad Reichenhall Clinic. Inclusion criteria include a referral diagnosis for uncontrolled asthma, no cognitive impairment and no very severe co-morbidities that indicate significantly greater illness morbidity than asthma alone. In the intervention group (IG), participants will start PR within 4 weeks after randomization; participants of the control group (CG) will start PR 20 weeks after randomization. Data will be assessed at randomization (T0), after 4 weeks (T1; IG: begin of PR), 7 weeks (T2; IG: end of PR), and 20 weeks (T3, CG: begin of PR). The primary outcome is asthma control at T2/T3. Secondary outcomes are health-related quality of life, functional exercise capacity, dyspnea, anxiety, depression, subjective self-management skills, illness perceptions, sick leave and subjective work ability. Outcomes will be analyzed with analysis of covariance, including baseline values of the respective outcomes as covariates. Healthcare costs will be analyzed with a gamma model with a log-link.

A longitudinal follow-up study will generate additional data at 3/6/9/12 months after PR for both IG and CG. Latent change models will be used to analyze the course of the primary and secondary outcomes. Annual cost differences before and after rehabilitation will be compared by paired t-test.

**Discussion:**

This RCT will determine the effectiveness of a complex inpatient PR for asthma patients concerning asthma control. Furthermore, important medical and economic information regarding the effectiveness of PR as part of the long-term management of patients with uncontrolled asthma will be generated.

**Trial registration:**

German Clinical Trials Register (DRKS00007740, May 15, 2015). Protocol version: 1.0 (December, 23, 2016).

## Background

### Introduction

Asthma is one of the most common chronic diseases worldwide with an estimated 300 million affected individuals [1]. Asthma was defined by the Global Initiative for Asthma (GINA) as a heterogeneous disease, usually characterized by chronic airway inflammation and a history of respiratory symptoms that vary over time and in intensity, including wheeze, shortness of breath, chest tightness and cough, together with variable expiratory airflow limitation [1]. Thus, the diagnosis of asthma is primarily based on clinical symptoms [2]. In spite of the availability of effective medications, it remains incurable. Various cross-sectional studies have shown a high number of patients with poorly controlled asthma in many countries [3, 4]. Therefore, new approaches to improve asthma control (AC) are urgently required.

Pulmonary rehabilitation (PR) is widely accepted as an effective treatment for patients with chronic respiratory diseases, especially for chronic obstructive pulmonary disease (COPD) [5]. In Europe and North America, asthma patients are also commonly enrolled in PR programs [6], but the available data regarding the effectiveness of PR for asthma patients are sparse. Only very few randomized studies [7, 8] concerning the effectiveness of a multimodal PR program in asthma have been published. All had certain methodological limitations, so that they weren’t considered for international evidence-based asthma guidelines [1, 2].

However, the effectiveness of the essential individual components of PR, such as patient education [9, 10], respiratory physiotherapy [11, 12] and aerobic exercise training [13–18] has been shown in several randomized controlled trails (RCTs). As a complete PR program is expected to be at least as effective as these single components, the German National Disease Management Guideline [19] recommends PR for asthma patients if physical, mental or social consequences of the illness are constraining and persist during daily life despite adequate medical therapy.

Besides the two RCTs [7, 8], a limited number of observational studies have been published in the international [20–24] and German literature [25–27]. They have shown positive effects regarding quality of life, clinical symptoms, physical function, exacerbations, and health care resource utilisation. However, up to now, no RCTs have addressed the question whether and how long asthma control improves after PR. Therefore, such a study was strongly recommended by the German National Disease Management Guideline [19].

This lack of evidence is of particular relevance since asthma is a common indication for PR in Europe and in the US [6], and is the most frequent indication for PR in Germany [28]. Therefore, the main objective of the EPRA-RCT is to fill the knowledge gap for PR in asthma. Moreover, there is little empirical data regarding the long-term course of AC, quality of life and self-management skills after PR for asthma and the cost-effectiveness. As part of the EPRA study a follow-up assessments at 6, 9 and 12 months after PR will be provided.

### Study aims

The primary aim of this RCT is to evaluate the effectiveness of PR for patients with asthma on short (end of rehabilitation) and intermediate-term (3 months after rehabilitation) outcomes as compared to a waiting-list control group. We hypothesize that pulmonary inpatient rehabilitation is superior regarding mean change in AC at 3 months after rehabilitation (primary outcome). Furthermore, we expect superior effectiveness of the pulmonary rehabilitation regarding health related quality of life (HRQoL), functional exercise capacity, dyspnea, anxiety, depression, subjective self-management skills, illness perceptions, sick leave, as well as subjective work ability (secondary outcomes). In addition, moderator effects of (a) gender, (b) age, (c) baseline AC, (d) baseline HRQoL and (e) baseline anxiety will be examined. Moreover, in a longitudinal follow-up study, the course of primary and secondary outcomes over one year and the annual costs before and after rehabilitation will be explored.

## Methods

### Study design and data collection

We use a two part-study design, combining a randomized controlled trial (RCT) and a longitudinal follow-up design.

The main study is a randomized control superiority trial, comparing the intervention group (IG) with a waiting-list control group (CG). For legal reasons, any insurant of the statutory German Pension Insurance (GPI) whose application for medical rehabilitation was approved by the GPI will receive a rehabilitative intervention. Therefore, it is not possible to use an entirely untreated CG, but a waiting-list CG can be realized. After inpatient rehabilitation in the Bad Reichenhall Clinic has been approved by the GPI, patients receive a letter informing about the study and screening for their eligibility. If no response is received within 14 days, a reminder will be sent to the patients. If patients meet the inclusion criteria, they are asked to participate. After consent has been obtained, participants will be randomized to the IG or CG. In the IG, participants will start inpatient rehabilitation within 4 weeks after randomization. Data will be assessed at randomization (T0), at the beginning (T1), the end (T2), and three months after inpatient rehabilitation (T3). The CG will start inpatient rehabilitation five months (i.e. 20 weeks) after randomization. CG data will be assessed at randomization (T0), at four weeks (T1), at seven weeks (T2) and at 20 weeks (T3, beginning of inpatient rehabilitation) after randomization. The crucial reference intervals for the primary research questions are T0 to T2 and T0 to T3 for both IG and the CG. Since CG starts PR 19 to 20 weeks after T0, which is 3 months after the discharge time-point of IG, it will be possible to compare the results of IG with those of the (still) untreated CG during these initial 19 to 20 weeks. However, it may be possible that for the CG, arrival at the rehabilitation clinic (at T3) already has an effect on several outcome parameters. To estimate this “arrival effect”, an additional measurement time point (T3a) for the CG at 10 (+/-3) days before T3 has been included.

**Fig. 1:**
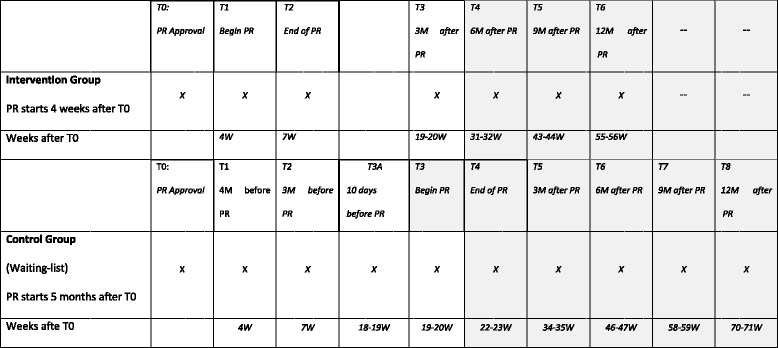
Study design and measurement occasions; PR: pulmonary rehabilitation; M: months; W: weeks; not shadowed: data for randomized control trial; grey shadowed: data used only in the longitudinal study

The second part of the study is a longitudinal cohort study. In addition to the data collected in the first part of the study (RCT), data at 6, 9 and 12 months after inpatient rehabilitation in the IG and data at the end of and at 3, 6, 9 and 12 months after inpatient rehabilitation in the CG will be collected. Longitudinal data from both groups will be combined to one sample. Fig. 1 illustrates the study design. Note that measurement time points of the IG and CG have a parallel timing only from T0 to T3. Participants not returning the postal questionnaires on time will be reminded by phone. If necessary, questionnaires will be sent again by mail to the patient.

### Patient selection

Patients are eligible for the study if they are approved for PR, have a physician diagnosis of asthma (ICD-10: J45) at T0 and have uncontrolled asthma based on an Asthma Control Test (ACT) <20 [1, 29, 30]. Patients will be excluded if they have cognitive impairment, inadequate German language ability, or severe concomitant diseases that might mask the results of asthma rehabilitation (for example cancer and severe cardiac/orthopedic or psychiatric comorbidities). The initial diagnosis of asthma will be confirmed by a pulmonologist at admission to inpatient rehabilitation. If the initial diagnosis cannot be confirmed, the patient will be excluded from the study.

### Randomization

Participants will be recruited consecutively. All patients meeting the inclusion criteria and providing informed consent are randomly assigned to the IG or CG by a study nurse at the time of their PR approval. Randomization will be stratified according to age. The randomization list (with computer-generated random numbers) is created by the Department of Medical Psychology and Psychotherapy, Medical Sociology and Rehabilitation Sciences at the University of Würzburg (concealed allocation).

### Blinding

Patients themselves cannot be blinded due to the time-point of the start of their inpatient rehabilitation. However, those who deliver the rehabilitation treatment are unaware whether the patient is a study participant, a participant of the IG or the CG, or a regular inpatient outside the study.

### Sample size

Calculation of sample size is based on our primary outcome, the ACT, assessed three months after the inpatient rehabilitation. A previous longitudinal study of asthma patients in the Bad Reichenhall Clinic (without a CG) showed changes between start of inpatient rehabilitation and three months after inpatient rehabilitation of (Cohen’s) d > 0.5 [25]. However, this value might overestimate the effect of inpatient rehabilitation compared to a control group. Therefore, sample size was calculated to detect a difference of d = 0.3 at T3 between the IG and CG. Using alpha = 0.05 and power = 0.8, 352 participants are required, but assuming a drop-out rate of 30%, a total of n = 504 patients will be recruited [26].

Latent curve models will be used to analyze the course of the primary outcome in the longitudinal follow-up study. As a rule of thumb, N > 100 cases should be included in the computation of these models. Even with a conservatively estimated drop-out rate of 50% until T6 for IG and T8 for CG, N = 152 patients would remain in the study, which is sufficient.

### Intervention

As shown in Fig. 1, both groups receive a 3-week PR that meets the structural requirements of German healthcare insurance providers [31, 32]. The PR is carried out by a multi-professional team (physicians, psychologists, physiotherapists, sports scientists, social workers, nutritional consultants) and will be tailored individually to each patient’s needs. The rehabilitation program will be reviewed at least once a week as part of the doctor’s weekly rehabilitation ward round.

The rehabilitation program includes the following non-drug therapy components (O = obligatory for all participants, except those with individual contraindications, F = facultative if needed):

1. Physical training (O) consisting of three obligatory components: a) endurance training scheduled for 5 units per week for 45–60 min each, during which outdoor sports and training in water (e.g. Aqua Fitness, Nordic Walking) are performed. Exercise intensity is controlled by BORG Scale and heart rate; b) strength training scheduled for 3 sessions per week of 45–60 min each and c) whole-body vibration-training scheduled 7 times per week. In addition, inspiratory muscle training (F) is provided for patients with inspiratory muscle weakness and is scheduled for 7 sessions per week, each for 21 min, of which 2 per week are supervised.
2. Comprehensive patient education (O) consisting of two obligatory components: a.) patient education regarding asthma for one week with 7 units of 45 min; b.) one session of practical medical inhalation training and/or peak flow meter use for 60 min. In addition, patients also receive allergy awareness and trigger avoidance training (F) if required (one 60-min session).
3. Respiratory physiotherapy (O) consisting of 3 units of group respiratory physiotherapy per week with learning of pursed lips breathing (O) for 45 min each. If necessary, patients may also receive the following optional components (F) including: a) individual breathing training by physiotherapists; b) training of Buteyko breathing techniques [29] for patients with dysfunctional breathing [30]; c) physiotherapy seminar on coughing techniques for patients with cough problems; d) mucolytic inhalation therapies (e.g. saline inhalation, for patients with mucostasis (4).
4. Psychosocial support (F) such as social counseling, individual psychological counseling and/or group therapy will be offered if necessary (Patient Health Questionnaire (PHQ-9) > 9 points, Generalized Anxiety Questionnaire (GAD-7) > 9 points or if indicated by their physicians).
5. Comprehensive smoking cessation program (F) will be offered to all smokers.
6. Comprehensive nutritional counseling (F) will be offered to patients with food intolerance or allergies or over- and underweight.

In addition, patients will receive a routine check-up and if necessary, current asthma medications will be optimized according to the current guidelines. This is an obligatory part of the rehabilitation in Germany. Any changes to medications will be documented. Adverse events and complications during rehabilitation are recorded by the physicians on a standardized basis in the medical survey sheet at the end of rehabilitation.

### Outcomes and measures

The primary outcome is the mean change in AC, assessed by ACT. Secondary outcomes include mean changes in HRQoL, 6-min walk distance (6-MWD) and Sit-to-stand-test, lung function parameters, Eosinophils, dyspnea, depression, anxiety, smoking habits, illness representations, self-management skills, work ability, subjective prognosis of return to work, sick leave, medication beliefs and medication adherence. Measurement instruments for all outcomes and measurement occasions can be found in Table 1. Lung function parameters, 6-MWD, FeNO, and Eosinophils are assessed at the start and the end of the inpatient rehabilitation by staff of the PR. All other outcomes are assessed via questionnaires.

**Table 1:** outcome measures

Outcome	measured by/Instrument	T0	T1	T2	T3A (CG)	T3	T4	T5	T6	T7 (CG)	T8 (CG)
Asthma control	Asthma control test (ACT) [29, 30]	X	X	X	X	X	X	X	X	X	X
Lung function, blood gases	FEV1, FEV1/VC, SRtot, VC, RV, PaO2, PaCO2, [50, 51]		IG	IG		CG	CG				
Exercise capacity	6MWT [53]										
Allergy	Total Ig E, specific Ig E-Screening (ImmunoCAP®)		IG	IG		CG	CG				
Asthmatic inflammation	Fractional exhaled nitric oxide (FeNO) [56], Eosinophils		IG	IG		CG	CG				
QoL	Saint George’s Respiratory Questionnaire (SGRQ) [35]	X	X	X	X	X	X	X	X	X	X
QoL	Asthma Quality of Life Questionnaire (AQLQ)] [36]	X	X	X	X	X	X	X	X	X	X
QoL	EQ-5D-5L [37, 38, 57]	X	X	X	X	X	X	X	X	X	X
QoL	Global Rating of Change Scale (GROC-scale) [39]			X	X	X	X	X	X	X	X
Symptoms	Numerical rating scale for dyspnoea, cough, sputum, pain [40]	X	X	X	X		X	X	X	X	X
Dysfunctional breathing	Nijmegen Questionnaire (NQ) [58]	X	X	X	X					X	X
Depression	Patient Health Questionnaire (PHQ) [59]	X	X	X	X		X	X	X	X	X
Anxiety	Generalized Anxiety Disorder Questionnaire (GAD 7) [60]	X	X	X	X		X	X	X	X	X
Fatigue	Brief Fatigue Inventory (BFI) [41]	X	X	X	X						
Illness Perception	Illness Perception Questionnaire (IPQ-R) German Version [45]	X	X	X	X		X	X	X	X	X
Resource Use	FIM-Lu, modification of FIMA [52]	X			X		X	X	X	X	X
Work ability	Work Ability Index (WAI) Items 1 and 4 [61]	X	X	X	X		X	X	X	X	X
SPoRTW	SPE-Scale [55]	X	X	X	X		X	X	X	X	X
Self-management	Health Education Impact Questionnaire (heiQ), Scale “Skill and technique acquisition”	X	X	X	X		X	X	X	X	X
Smoking habits	Questionnaire (developed)	X			X		X	X	X	X	X
Sport behaviour	Questionnaire (developed)	X			X		X	X	X	X	X
Medication beliefs	Brief Medication Questionnaire (BMQ) [62]	X	X	X	X				X	X	X
Adherence	Medication Adherence Report Scale (MARS) [63]	X	X	X	X				X	X	X
Sick leave	Questionnaire (developed)				IG		X	X	X	X	X

Notes: QoL: Quality of Life; SPoRT: Subjective prognosis of return to work; IG: assessment only in intervention group; CG: assessment only in control grou

### Assessment of outcomes

#### Asthma control

Asthma Control will be assessed by using the Asthma Control Test (ACT) [30, 33]. The ACT consists of five items that assess (1) activity limitation, (2) daytime shortness of breath, (3) awaking due to asthma symptoms, (4) needed puffs of reliever medication and (5) a global judgment of asthma control. All items refer to the last 4 weeks. They are scaled from 1 to 5. The sum of scores indicates asthma control. An ACT score of 20 – 25 indicates controlled asthma and of <20 indicates uncontrolled asthma. A minimal important difference about 3 was identified [34].

#### Health related quality of life

Asthma specific HRQoL will be assessed by both, the Saint George’s Respiratory Questionnaire (SGRQ) [35] and the standardized version of the Asthma Quality of Life Questionnaire (AQLQ) [36]. The SGRQ uses 50 items to capture the three domains *Symptoms*, *Activity* and *Impacts* as well as a *Total* scale. All scales are computed by weighted sums of the respective items. The scores range from 0 (no impairment) to 100 (maximum impairment). A minimal important difference (MID) of 4 is established for COPD, a MID for asthma has not been reported up to now. The standardized version Asthma Quality of Life Questionnaire (AQLQ) contains 32 questions to capture the four domains *Symptoms*, *Activity limitation*, *Emotional function* and *Environmental stimuli* to measure the functional problems that are most troublesome to adults with asthma. The survey period covers the past 2 weeks. Each question is answered on a 7-point scale (1 = severely impaired - 7 = not impaired at all). The overall AQLQ score is the mean of all 32 responses and the individual domain scores are the means of the items from those domains.

Generic HRQoL of patients is measured using the Euroqol questionnaire (EQ-5D-5L) and the EQ visual analogue scale (VAS). The EQ-5D-5L [37] is a standardized instrument applicable to a wide range of health conditions for use as a measure of health outcome. It is especially suited to cost effectiveness analyses as it can be used to generate quality-adjusted life years [38]. The descriptive system of the EQ-5D-5L comprises five dimensions (mobility, self-care, usual activities, pain/discomfort, anxiety/depression). Each dimension has five levels: no problems, slight problems, moderate problems, severe problems, and extreme problems. A German utility index is currently being developed.

#### Subjective health

Global rating of change (GRC) [39] in subjective health is assessed using a single item that compares current subjective health with subjective health at the beginning of the inpatient rehabilitation. The response scale ranges from -7 (much worse) over 0 (no change) to 7 (much better).

#### Symptoms

Severity of dyspnea, cough, sputum and pain will be assessed with response to seven 11-point numeric rating scales [40]. Scale values range from 0 (no symptoms) to 10 (worst imaginable symptom severity).

#### Fatigue

The Brief Fatigue Inventory (BFI) will be used to measure fatigue. This short questionnaire assesses severity of and impairment from fatigue with ten questions. The subscales and the total score range from 0 to 10, with higher values indicating higher severity/impairment. A German version exists and has been proven reliable and valid [41].

#### Depression and anxiety

The PHQ-9 and the GAD-7 will be used to assess depression [42] and anxiety [43, 44]. All items are scored on a 4-point Likert scale (0 = not at all, 1 = several days; 2 = more than half of the days; 3 = nearly all days). Besides using the sum of scores (which range from 0 to 27 in PHQ and 0 to 21 in GAD-7), we will also classify individuals with values of >10 as indicating a clinically relevant depressive disorder (PHQ-9) or a clinically relevant anxiety disorder (GAD-7).

#### Illness representation

The 9-item Brief-Illness Perception Questionnaire (B-IPQ) [45] will be used to capture 8 aspects of *illness representations* (i.e how illness perceived by the patient: *Consequences*, *Timeline*, *Personal control*, *Treatment control*, *Identity*, *Concern*, *Understanding* and *Emotional response*. Each aspect is assessed by one item, except for *Emotional response*, which is assessed by two items. All items use a 0 to 10 response scale. Furthermore, perceived causes of asthma attacks are assessed via an open-ended response item, which asks for the three most important causal factors of their illness.

#### Subjective self-management

Subjective self-management will be assessed using the *Skill and technique acquisition* scale from the Health Education Impact Questionnaire (heiQ^TM^, [46, 47]). The items are scored on a 4-point response scale (1 = strongly disagree, 2 = disagree, 3 = agree, 4 = strongly agree). The overall score is computed as the unweighted mean of all items, with higher values indicating a higher subjective judgement of self-management.

#### Medication adherence and medication beliefs

Patients will complete the Medication Adherence Report Scale (MARS-D) [48]. This is a 5-item questionnaire with a 5-point response scale (1 = always to 5 = never). The sum of the individual answers can range from 5 to 25 points, with higher values indicating better medication adherence. The 10-item Brief Medication Questionnaire (BMQ) [49] will be used to assess patient’s medications beliefs on two scales. The scale *Necessity* assesses patients’ beliefs about the necessity of prescribed instruments and the scale *Concern* assesses patients’ concerns about prescribed medications. Both scales range from 5 to 25 points with higher values indicating higher belief of necessity/higher concerns.

#### Lung function measurement

Forced expiration in one second (FEV_1_), vital capacity (VC), residual volume (RV) and total specific resistance (SRtot) are determined using spirometry and body plethysmography (MasterLab, CareFusion, Hoechberg, Germany) before and after bronchodilation with a short-acting bronchodilator in accordance with recommendations of the national guidelines [50, 51].

#### Resource use

Resource use will be determined based on answers to the FIMA-Lu questionnaire, a modified Version of the FIMA questionnaire, a German instrument to assess health-related resource use [52]. For direct costs, the number of visits to a general practitioner, specialist care, ambulatory clinics in hospital, physiotherapy, days spent in hospitals and intensive care units and medication administered will be documented. For indirect costs, work absenteeism days will be documented.

#### Exercise Capacity

The 6-MWD will be measured using a track length of 30 m according to the European Respiratory Society/American Thoracic Society technical standards [53]. At T0 and T1 respectively, each patient performs the test twice with an interval of one hour. The best test will be included for analysis.

#### Work ability

Subjective work ability will be assessed by the first item of the Work Ability Index (WAI), the *Work Ability Score* (WAS). It compares current subjective work ability to the lifetime’s best. The 11-point scale ranges from 0 (complete incapacity to work) to 10 (lifetime’s best work ability). The WAS shows high correlations with the overall WAI score [54]. Furthermore, the 4^th^ Item of the WAI is used to assess health-related limitation of current work ability, with values ranging from 1 (total incapacity to work) to 6 (no limitation). In addition, subjective prognosis of employment status will be assessed using the 3- items of the Subjective Prognostic Employment Scale (SPE Scale). The three items assess patient’s belief (a) to remain at work until retirement and (b) whether their health will be permanently jeopardized and (c) whether they are considering applying for a disability pension [55].

#### Sport activity, smoking habits and medication use

Two items will be used to assess sport activity. In Item 1, the patient indicates whether he or she exercises regularly (at least 2 times per week) and in item two the kind of exercise performed (e.g. gym, sports club, lung sport group). Current smoking status (current smoking yes or no) and number of cigarettes per day will be documented. Furthermore, patients’ use of antibiotics or cortisone in the last three months (yes/no; number of uses) will be documented.

#### Socio-demographic data and employment

Information regarding socio-demographic data and employment status will be collected.

##### Data management

Data of all measurement time-points will be collected in the Bad Reichenhall Clinic. Questionnaire data will be entered into Microsoft Excel by two study nurses (independent double data entry by two individuals). A unique ID number will be assigned to each patient and personal data (name, address) will be stored in a completely separate file. The list with assigned ID numbers and personal data will be stored in the Bad Reichenhall Clinic and will not leave the center. Other anonymized data will be sent to the University of Wuerzburg and the Helmholtz Zentrum Munich for further data management and data analyses.

### Statistical methods

Primary and secondary outcomes will be analyzed according to the intention to treat approach [56]. All randomized patients will be included in the analysis. Missing data fulfilling the assumption of missing (completely) at random will be imputed using multiple imputation procedures [57]. In addition, because it may be possible that some patients will not start with the inpatient rehabilitation or decide to drop-out of the study, we will also analyze results based on a “per protocol” approach [58, 59] including only patients remaining in the study until T3.

The analysis of the primary and secondary outcomes at T2/T3 will use analysis of covariance (ANCOVA) with treatment group as a fixed effect and baseline value of the respective outcome as a covariable. Adjusted mean differences and confidence intervals will be reported. In addition, moderating effects [60] of the following baseline variables on the primary outcome will be analyzed: ACT, gender, age, quality of life (assessed via SGRQ Total Score), anxiety (assessed using GAD-7) and depression (assessed using PHQ-7). For all models, statistical model assumptions (linearity, homoscedasticity, normality of residuals) will be tested. If model assumptions are violated, appropriate analysis methods (e.g. robust regression, log-transformation) will be used.

Latent change models will be used for exploratory analyses of the course of the primary and the secondary outcomes in the follow-up longitudinal study [61, 62].

### Economic evaluation

Data regarding resource utilization and work absenteeism will be documented retrospectively by the patient. A self-administrated questionnaire based on the FIMA questionnaire [52] has been developed specifically for patients with pulmonary problems. The monetary valuation of resource use will be based on the valuation rates calculated by Bock et al. [63]. Analysis of cost data will be performed with a model with a gamma distribution and a log-link to account for the skewed distribution of the data [64]. A 95% confidence interval for the cost difference will be estimated based on 1,000 bootstrap replications using the percentile method.

In the longitudinal follow-up study, the annual costs before and after rehabilitation will be compared by paired t-test. Alternatively, bootstrap resampling with 1,000 resamples will be used to compute p-values because cost differences may have a skewed distribution.

All statistical Analysis will be performed using SPSS [65], R [66] & SAS software [67].

## Discussion

Asthma is one of the most common chronic diseases worldwide with an estimated 300 million affected individuals [1]. Although effective medications are available, asthma remains incurable and poorly controlled for numerous patients in many countries [3, 4]. In Europe and in North America, asthma patients are commonly enrolled in PR programs [6] but the data available regarding PR of patients with asthma are sparse. This will be the first RCT to assess the effectiveness of a complex inpatient pulmonary rehabilitation regarding asthma control. The intervention conforms to the quality guidelines of the German Statutory Pension Insurance and follows national and international guidelines for pulmonary rehabilitation. Therefore, the results of this study may be generalizable. Furthermore, relevant medical and economic information will be generated for third-party payers, on which they can base their decisions regarding long-term management of asthma.

The findings of this study will be published in peer-reviewed journals and conference presentations.

## Abbreviations

6-MWD: 6-Minute walk distance
AC: Asthma control
ACT: Asthma Control Test
AQLQ: Asthma Quality of Life Questionnaire
BFI: Brief Fatigue Inventory
B-IPQ: Brief-Illness Perception Questionnaire
BMQ: Brief Medication Questionnaire
CG: Control group
COPD: Chronic obstructive pulmonary disease
EQ-5D-5L: Euroqol Questionnaire
FeNO: Fractional exhaled nitric oxide
FEV_1_: Forced expiration in one second
FEV_1_/VC: FEV1/FVC ratio, also called Tiffeneau-index
GAD: Generalized Anxiety Questionnaire
GRC: Global Rating of Change
heiQ: Health-Education Impact Questionnaire
IG: Intervention group
ImmunoCAP®: Measurement of specific IgE (Immunoglobulin E) antibody blood levels
HRQoL: Health related quality of life
MARS-D: Medication Adherence Report Scale
MID: Minimal important difference
PaCO2: partial pressure of arterial carbon dioxide
PaO2: partial pressure of arterial oxygen
PHQ: Patient Health Questionnaire
PR: Pulmonary Rehabilitation
QoL: Quality of Life
RCT: Randomized controlled trial
RV: Residual Volume
SGRQ: St. George Respiratory Questionnaire
SPE Scale: Subjective Prognostic Employment Scale
SPoRT: Subjektive prognosis of return to work
SRtot: Total specific resistance
Total Ig E: total Immunoglobulin E
VC: Vital capacity
WAI: Work Ability Index
WAS: Work Ability Score
